# An Infrastructure to Enable Lightweight Context-Awareness for Mobile Users

**DOI:** 10.3390/s130809635

**Published:** 2013-07-29

**Authors:** Pablo Curiel, Ana B. Lago

**Affiliations:** Deusto Institute of Technology-DeustoTech, University of Deusto, Avda. Universidades 24, Bilbao 48007, Spain; E-Mail: anabelen.lago@deusto.es

**Keywords:** context management, semantic technologies, pervasive computing, mobile computing, context-aware services

## Abstract

Mobile phones enable us to carry out a wider range of tasks every day, and as a result they have become more ubiquitous than ever. However, they are still more limited in terms of processing power and interaction capabilities than traditional computers, and the often distracting and time-constricted scenarios in which we use them do not help in alleviating these limitations. Context-awareness is a valuable technique to address these issues, as it enables to adapt application behaviour to each situation. In this paper we present a context management infrastructure for mobile environments, aimed at controlling context information life-cycle in this kind of scenarios, with the main goal of enabling application and services to adapt their behaviour to better meet end-user needs. This infrastructure relies on semantic technologies and open standards to improve interoperability, and is based on a central element, the context manager. This element acts as a central context repository and takes most of the computational burden derived from dealing with this kind of information, thus relieving from these tasks to more resource-scarce devices in the system.

## 1. Introduction

Mobile devices have experienced remarkable changes since their commercial appearance in the early 1980s. From the first models, which mimicked the functionality of traditional telephones but in portable format, mobile phones have steadily evolved integrating different capabilities like text messaging, network connectivity, full colour and resolution displays and diverse applications among others, until they have turned into the recent smartphones. This technological growth has also been accompanied by an important increase in the popularity of these devices. Thus, as of 2013 there are almost as mobile-cellular subscriptions as people in the world [1], having this number nearly doubled since 2008, when smartphones begun to become popular with the appearance of the first generations of iPhone and Android devices. This way, mobile phones have become everyday-use devices in which we rely on to carry out a great variety of tasks. Nevertheless, they still suffer from more processing power and interaction capability limitations than traditional computers. Indeed, the often distracting and time-constricted scenarios in where those interactions take place do not help in alleviating these limitations.

However, adapting and personalizing mobile applications to each situation and user can provide a great added value by simplifying and minimizing the required interaction. Among the different approaches to achieve this goal, context information is one of the most important in mobile devices [2]. In fact, current smartphones, thanks to the wide variety of sensors they are equipped with, their permanent network connectivity and the great diversity of applications they can execute are able to provide richer and better context information than ever before. By context we mean any information that can be used to characterize the situation of an entity (where entity is a person, place, or object) that is considered relevant to the interaction between a user and an application, including the user and applications themselves [3]. And using this information it is possible to create context-aware application and services which can provide personalized information, react to different situations and adapt their behaviour to better meet end-user needs [4].

Pursuing this goal, in this work we present an infrastructure for managing context information in mobile environments. This infrastructure is responsible for dealing with context information during its whole life-cycle, from the acquisition of this type of information to the usage of it in benefit of the user, including the intermediate processing needed to assure that it is available for all the entities that require it to successfully carry out their tasks as well as the suitable storage and maintenance of historical information. One of the key issues to be addressed by a context management infrastructure is guaranteeing a successful and efficient interaction among all the entities present in the environment. To achieve this goal, both a common model for information representation and for accessing and sharing it is needed. Our infrastructure relies on semantic technologies for this purpose, allowing an easier interoperability with other systems. On to other terms, as the main purpose of this work is to give service to resource-scarce devices like mobile phones, a central repository or blackboard approach [5] is adopted for the context information storage. Even though the computational power of mobile devices has remarkably increased in the recent years and continues to evolve, computation on them will always be a compromise, and they will necessarily be resource poor relative to static client and server hardware, as stated by Satyanarayanan [6]. Indeed, even if computational capabilities of mobile devices have noticeably increased, their batteries have not evolved at the same pace, and working with context information involves computationally demanding and intensive tasks, like semantic reasoning, which can compromise battery lifetime. Similarly, context provision in scenarios with a great number of consumers could imply having to answer to a high number of queries. And context consumption involves searching for providers that supply the needed context information and individually querying them. This way, the presence of this central element will allow mobile entities to work with context information in an easy and lightweight manner, as it takes most of the described computational burden derived from dealing with this kind of information. It also enables mobile devices to make the context information they capture available for the rest of the entities without needing to expose a querying endpoint to attend each context request, as it is the context manager the one who receives, stores and later serves it. And this also enables them to access the information they need without having to care about which context source or sources produce it and query each of them individually.

The remaining of this paper is structured as follows. In Section 2 related work is reviewed. Then, in Section 3 the context management infrastructure is described. Next, in Section 4 the implemented prototype is explained along with the validation scenario designed and the performance tests carried out. Finally, in Section 5 discussion and future work are exposed.

## 2. Related Work

In the recent years, numerous context-aware systems have been proposed in the literature, several of which are also targeted at mobile environments.

In [7] one of the first proposed context-aware architectures is described. Trying to ease the development of context-aware applications, they propose the concept of context widget, inspired in the GUI widgets, which mediate between the application and the environment. These widgets are comprised of three elements: generators that retrieve context information, interpreters that abstract it, and aggregation servers. Widgets can also be combined together and provide a context information subscription mechanism for the applications.

The SOCAM architecture proposal by Tao Gu *et al.* [4] tries to ease building context-aware mobile services. For this purpose, as our infrastructure does, they rely on an ontology model, enabling knowledge sharing and context reasoning. A locating service is also introduced, which allows discovery of both context providers and mobile services. To trigger context-driven behaviours they use a rule set that is fired whenever the context changes. And to allow mobile services access context information, they provide both pull and push modes. However, consumers need to look for context sources that provide the information they need and either query them or subscribe to their updates, while we adopt a data-driven approach that relieves consumers from the need of directly querying consumers.

The MOBE architecture by Coppola *et al.* [8] intends to send applications to mobile devices depending on the context the user is in. MOBE differs from our solution in that it delegates most of the computational burden to mobile phones, making them responsible for both gathering and processing context information, as well as deciding which applications to request in each case. It also restricts context information usage to select and adapt mobile applications, while in our system both mobile applications and applications executed in the network can take advantage of this information.

In [2] Gehlen *et al.* present a mobile Web Services framework. In an opposite approach to our centralized solution, they suggest a peer-to-peer strategy in which each mobile device publishes context information for others to access it. For this purpose and taking into account the limitations of mobile devices, they design ad-hoc XML and XSD-based languages both for context information sharing and for defining publish/subscription rules. Similarly, they adapt SOAP with UDP as communication protocol.

The SCOUT framework by Woensel *et al.* [9], aimed at easing the development of context-aware mobile applications, shares several similarities with our approach. However they specifically focus on the concept of nearby entities in the user environment, which can be discovered to provide relevant information and services to them. For this purpose, they bet on providing physical entities with Web presences which can be semantically queried. And in this way their approach is based on combining the information provided by the Web presences of these physical entities with users' own information stored in the mobile device to enable the mentioned personalization. However, this proposal limits the role of mobile devices to information consumers which query their nearby entities.

The framework for Android mobile devices proposed by Hu *et al.*, ContextTorrent [10], enables context storage and sharing among local and remote applications in a distributed peer-to-peer network. It employs an ontology-based approach, and provides context storage and querying capabilities inside the mobile device. However, considering the limitations of this kind of devices, it proposes an object-oriented database storage system for context information, thus limiting the semantic query expressiveness. Similarly, the context sharing among remote applications is thought as a peer-to-peer RDF triple sharing, rather than exposing a remote querying endpoint.

Kalimucho [11] is a platform for ubiquitous applications which enables automatic and personalized service deployment in mobile devices. For this task instead of relying on context information, they propose a device-resource based approach. This way, the platform creates and deploys adapted services into the devices and later monitors hardware resources like device battery or memory during their execution, thus ensuring an adequate QoS. It also enables users to define resource consumption preferences.

Iglesias *et al.* [12] presented a semantic reasoning infrastructure for mobile devices, aimed at simplifying the creation of context-aware applications. This framework provides a sensing subsystem, which abstracts access to both physical and virtual sensors, and a context management subsystem, which using an ontology-driven model fuses and reasons over this context information. Finally, a core subsystem enables an event-based communication mechanism to provide applications with context data. In a similar case to the MOBE architecture, this proposal differs from ours in that, apart from delegating context-management tasks to mobile devices, it does not consider an scenario in which mobile devices act as providers of context information for other entities in the environment.

The work by David *et al.* [13] proposes a middleware for Android devices for an easier development of context-aware mobile applications. It adopts a Context-Oriented Paradigm, which based in a context information publish-subscribe mechanism enables an agile application behaviour adaptation. However, with a special focus on interface adaptation, they limit the scope of context provision, management and consumption to sensors and applications of the mobile device itself.

Finally, in [14] a model and an architecture for context-aware mobile collaborative systems are proposed. The basic idea is that users, either alone or in collaborative manner, in each space and context, can assume certain roles and carry out activities with defined objects. To support this collaborative environment they rely on a distributed context management architecture, in which both a central server and end-user mobile devices store context information. This distributed approach, though, does not differ much from our solution, as context managed by mobile devices is mainly personal information for the usage of the device itself. Thus, more generic information which is by all entities in the environment, like the one related to groups and spaces, is stored in the central server.

## 3. The Context Management Infrastructure

In a context-aware system the context management infrastructure is a key component, as it is responsible for aggregating, storing and making context information available for the rest of the entities to successfully carry out their tasks. Its role is even more relevant in mobile environments, where the infrastructure has to take into account the limited capabilities of some entities in the system, enabling an easy and low-overload interaction with context information. In this section we describe the proposed architecture for context information management and the API exposed for the rest of the system to work with it.

### 3.1. Architecture

**Figure 1.**
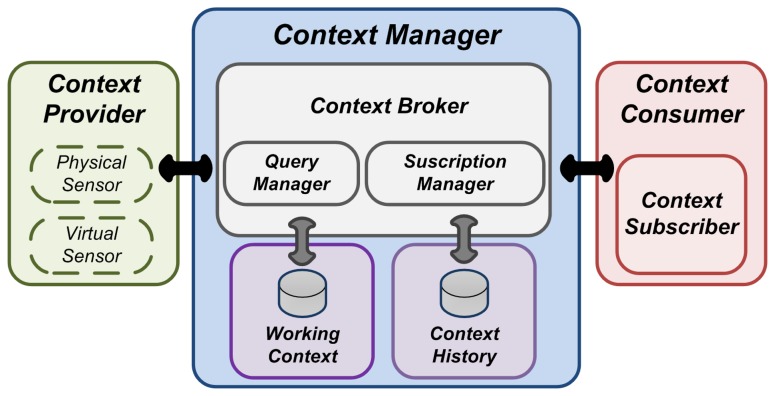
Context Management Architecture.

Depending on the tasks they carry out with context information, the elements that compose the context management architecture (see Figure 1) can be classified in three main groups: context providers, context consumers and the context manager. These components and their functionality are detailed below.

#### 3.1.1. Context Providers

A context provider (also known as context source) is any entity which provides context information to the rest of the elements in the environment. A context provider can be of a great variety of types: a physical sensor, an end-user mobile device, a social network, a mobile service, *etc.* Should be noted that the information managed in a context-aware system must be represented using a common knowledge representation model, so context sources must perform this information provision according to its requirements.

#### 3.1.2. Context Consumers

A context consumer is whichever entity that requires context information to perform different tasks, like information presentation, context processing for inference purposes, behaviour adaptation or decision taking. In our case, most common consumers are mobile application and services, which make use of context information to adapt their functioning to meet the needs of end-users. To support flexible scenarios, consumers can access context information both synchronous and asynchronously. For the second approach a special type of consumer is defined, the context subscriber.

##### Context Subscriber

This type of consumer implements a standardized interface which enables it to asynchronously receive context information.

#### 3.1.3. The Context Manager

The context manager is the central element of the context management infrastructure. As said, mobile devices, even if its processing power has noticeably increased in the last years, are yet more computationally-limited than conventional computers, and thus have greater limitations to carry out demanding tasks with context information. Therefore, the context manager is introduced, as an element that provides centralized access to context information and which takes most of the computational burden of dealing with this kind of information, and so alleviating this problem. This approach has also other advantages in terms of work offloading from resource-limited devices. As the context manager exposes methods for sources to provide context information and for consumers to access it, independence between these two entities is guaranteed, avoiding the need for a direct communication between them. Indeed, context sources are relieved from the need of exposing a query interface. On the other hand, a data-centric approach is implicitly adopted for context access, as consumers request the information they need directly to the context manager, which has previously got it from the sources, and thus they only need to care about what information they need, not where to retrieve it from. However, the context manager is not designed as an atomic entity that carries out all the context management tasks itself. Thus, it is comprised of various independent components, each of them in charge of performing different tasks with context information, favouring both reuse and scalability. These components are the following.

##### Current Context

This element stores the context information which is valid in each moment, that is, the one that represents the current status of the entities which are considered to be part of the context. As it has been laid out, the information managed by the system must be represented and shared using a common model known by all the entities in the system. More precisely, ontologies are used for this purpose, as they are considered the best solution for context modelling in ubiquitous computing applications [15], due to their ability to deal with uncertainty, flexibility to integrate data coming from different sources and reasoning capabilities among other reasons. So the storage and access provided by this component are ontology-based.

##### Context History

In contrast to the current context, which only stores currently valid information, the context history keeps track of the changes taken place in the context information. This way, when a context information item changes, its earlier state together with its corresponding timestamp is registered in this repository. For instance, when users change their current location, their previous one is stored in the context history. This allows to later check changes on this attribute over time. On the other hand, depending on the strategy followed for the historical information storage, this entity could require a great amount of space. Hence, several policies have been established, such as the number of entries to store, information time-to-life, which entities should be stored and which not,… so as to enable different configurations for each specific scenario.

##### Context Broker

This component is responsible for managing both access to current and history context repositories. This way, it stores in the current context the context information received from the sources, moves outdated information from the current context to the context history, and attends consumers' requests querying these two repositories. At the same time, in order to comply with the requirement of providing consumers both with support for synchronous and asynchronous access modes to the context information, it has two subcomponents: the query manager and the subscription manager.

##### Query Manager

This element is responsible for answering synchronous context information requests which consumers make.

##### Subscription Manager

This component enables consumers to receive asynchronous notifications when given conditions are satisfied by the current context. For this purpose, consumers register subscriptions, which are comprised of the mentioned conditions and a callback address to be notified when those are satisfied. The subscription manager stores this subscriptions and whenever the current context is modified checks whether their conditions are satisfied in order to notify the corresponding consumers.

### 3.2. The Context Management API

Even though the context manager is comprised of a series of components, it provides a unique interface for the rest of the entities in the system to work with context information, the context management API. This also enables supporting different communication protocols to interact with the context manager, as this interface is merely an access layer, belonging the operation logic to the internal components exposed in the previous section. The context management API exposes several methods to manage context information which are independent from the underlying model and rely on standard technologies like RDF [16] and SPARQL [17]. Among those, the following can be emphasized as being the most relevant ones:

- *Add Context Information.* This method enables providers to perform add and update operations against the current context by providing information consistent with the ontology model and in RDF format.
- *Remove Context Information.* Using this method enables providers to delete an instance from the current context given its URI.
- *Get Context Information.* This method enables consumers to retrieve a known instance from the current context given its URI.
- *Query.* By calling this method, a context consumer can synchronously access the current context space using SPARQL queries.
- *Query History.* In a similar way the previous method enables consumers to access the current context, this allows them to perform SPARQL queries against the context history.
- *Subscribe.* This method enables a context consumer to asynchronously access the current context space. The consumer provides a condition and a callback address through which he wants to be notified when this condition is matched. This condition can be of two kinds: either an SPARQL query to be satisfied or a template consisting of a set of RDF triples to be matched by the current context.
- *Notify.* This method is not exposed by the context manager itself, but by those consumers which make use of the asynchronous subscription flow. This way, each time an asynchronous query is satisfied, the context manager invokes this method of the corresponding consumer using the callback address supplied in the *Subscribe* method.
- *Unsubscribe.* The context manager keeps track of the context information that fires a subscription, in order to avoid the same subscription being fired by the same data more than once. However, consumers may want to stop receiving context subscriptions at any point in time. For this to be effective, a consumer must provide the subscription ID which corresponds to the subscription that wants to remove.

## 4. Implementation and Evaluation

In this section we describe the implementation of the context manager which follows the design detailed in the previous section. A demonstration scenario is also presented, in which context source and consumers are involved in form of a mobile end-user application and a contextual service. A series of tests carried out to assess the performance of the infrastructure are also explained.

The prototype involves a fully functional context manager developed in Java and using the OSGi [18] component framework. To work with context information we use the semantic web toolkit Jena [19]. This library enables us to represent context information as an RDF graph, providing either in-memory or persistent storage (using Jena TDB) schemes. It also supports to define ontologies which describe the structure of this RDF graph and which enable reasoning over it. This way, current context is defined as an in-memory repository with support for on-line reasoning and context history as a persistent triplestore. On the other hand, Jenabean [20] library is also used, as it offers a POJO-to-RDF mapping which eases the interaction between Java objects and Jena RDF graphs. Regarding the context manager API, it is exposed as a RESTful [21] interface, which has gained popularity in the last years due to its simplicity an lower overhead compared to other approaches like SOA.

### 4.1. Demonstrative Scenario

**Figure 2.**
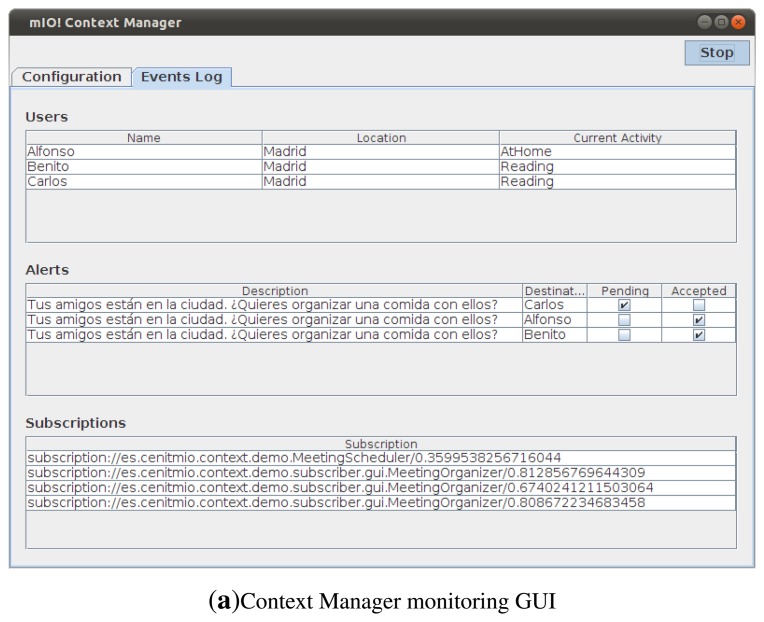

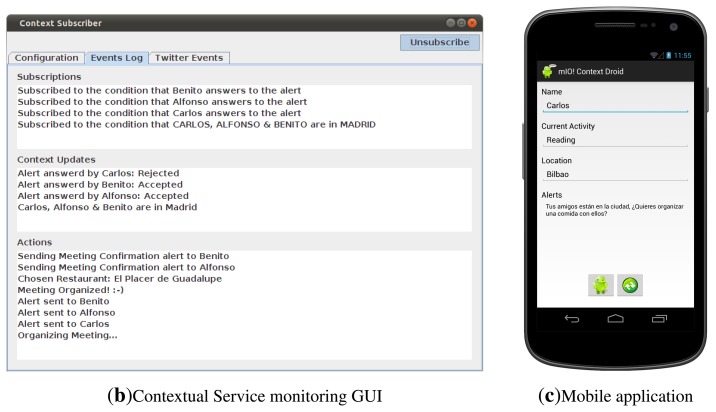
Prototypes of the Context Manager (a); Contextual service (b) and Mobile application (c).

**Figure 3.**
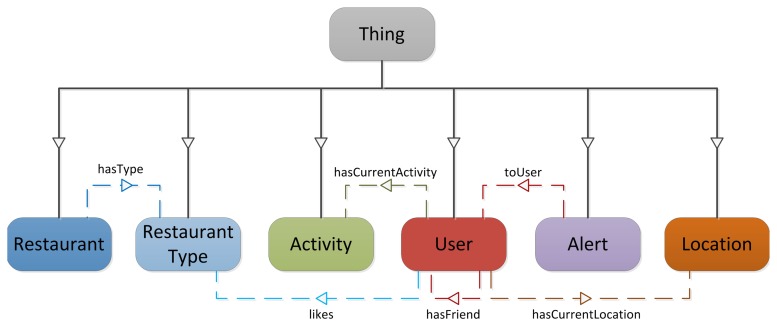
General Ontology.

**Figure 4.**
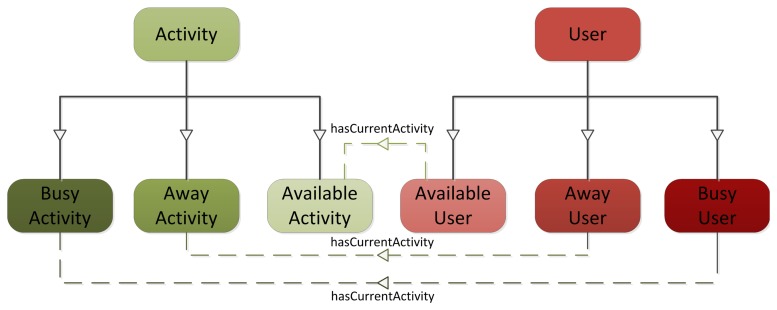
Users and Activities Ontology.

As a first validation step, a demonstration scenario was implemented. This scenario involves an end-user Android application (Figure 2(c)) and a contextual service (Figure 2(b)) interacting with the context manager (Figure 2(a)). The ontology shown in Figure 3 was also designed to represent the context of this scenario. This ontology models information about Users, Restaurants and Restaurant Types that users like, Locations where users are, Activities users are involved in and Alerts which are sent to users to inform them about plans with their friends. The aim of this scenario is to suggest available users to meet their nearby friends. For this purpose, the contextual service needs to know both users' location and their availability. Users availability is inferred from their current activity. The contextual service monitors the Twitter accounts of the users and from the messages they post in this social network, it extracts their current activity. Performing natural language processing to extract activities from user messages is out of the scope of this work, so for the scenario fixed messages are mapped to the different activities shown in Figure 4 (e.g., the message ‘I’m going to the cinema to watch Sharknado' would be mapped to AwayActivity and the message ‘I’ve got a long meeting this morning' would be mapped to BusyActivity). Then, as it can be observed in Figure 4, the ontology relates each type of activity to a user availability (e.g., an AvailableUser is defined as a subclass of User which has an AvailableActivity as current activity), so this information is automatically inferred by the semantic reasoner. To keep track of users' location, the contextual service registers subscriptions in the context manager to know when various users are nearby. So, when it detects friends in the same location (in this case location is equal to city, though this could be configured as desired), it queries the context manager to check if they are available, and if so sends them an alert suggesting a meeting for a meal. On the other hand, the end-user application acts as context source of users' location, keeping the context manager up to date of its changes. It also acts as user interface of the contextual service, querying the context manager in search for alerts which suggest plans to the users and enabling them to accept or decline those.

Following, a possible real use example of this scenario is shown. Freddie and Roger, who live in Bilbao, and Brian, who lives in Madrid, are three friends who use the “Social Alerts” service. One weekend Brian travels to Bilbao, and as a result the context manager notifies the service of this fact. Unfortunately, Roger has a busy weekend, and has told his Twitter followers about that fact with the tweet ‘This weekend will be no fun! Gotta have a report for Monday morning…’So the service, that periodically checks the Twitter accounts of the three friends, concludes that his activity is a BusyActivity. Thus, when it receives the notification from the context manager and checks the status of the three friends, it is inferred that Roger is not available. So it creates alerts for Freddie and Brian suggesting a plan. If both accept the alerts, the service queries the context manager to know their favourite restaurant types and selects one that they both like to organize the lunch.

### 4.2. Performance Tests

Starting from the described demonstrative scenario, a series of tests were carried out to assess the performance of the proposed infrastructure. These tests were aimed at evaluating the most demanding operations: context provision by the sources, context requesting by the consumers and reasoning. Thereby, the contextual service and the mobile application described in the previous section assume these roles in the following four scenarios:

(A) The service adding instances of the Alert class (see Figure 3), at a rate of two alerts per second.
(B) The service adding alert instances and the mobile application performing synchronous queries for those alerts, both concurrently and at a rate of two operations per second.
(C) The service adding alert instances at a rate of two alerts per second, being the mobile application subscribed to asynchronously receive them.
(D) The mobile application updating the user profile (an instance of the User class shown in Figure 3) at a rate of two updates per second. These updates were tested with the context history in different configurations: disabled, enabled with no filters (all changes are registered) and enabled with a filter (only changes that imply the current activity of a user being modified are registered).

As an example, an alert instance added by the service in RDF/XML format and a SPARQL query used by the mobile application to retrieve that alert are shown in Listings 1 and 2.

The main goal of these tests is to assess the response of the infrastructure to an increase in both the amount of context information and in the number of concurrent source and consumers. Therefore, measures were taken for the different tests with a range of 0 to 10,000 instances (or 0 to 60,000 triples, as both the added alert instances and the updated user instances consist of 6 triples) in the current context and the context history. At the same time, the tests include evaluating each of these scenarios with a different number of context source and consumers concurrently interacting with the context manager. The different numbers of clients chosen were: 1 client, 4 concurrent clients (one for each CPU core of the testing machine) and 8 concurrent clients (two per CPU core).

**Figure 5.**
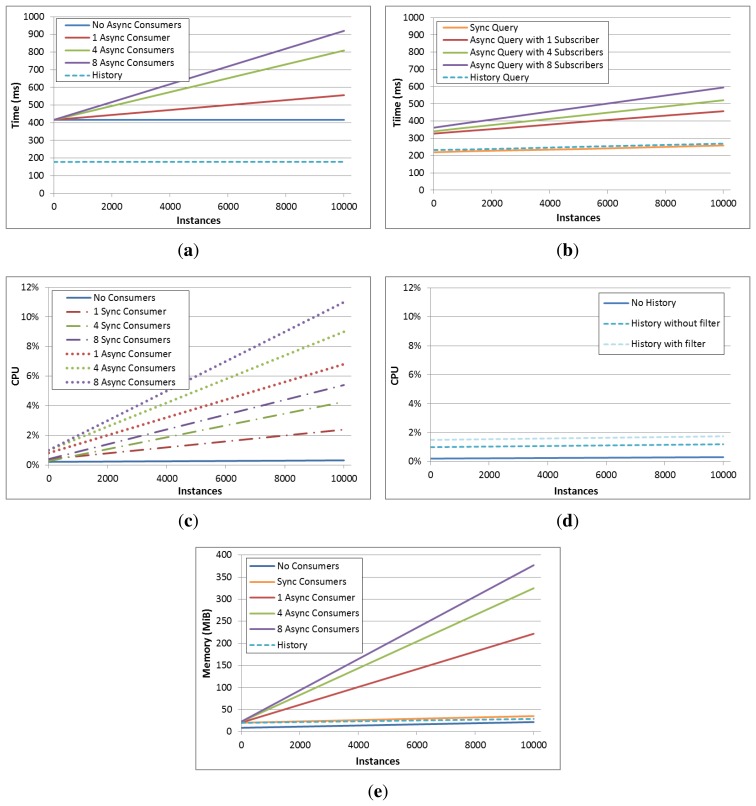
System performance in the different testing scenarios. (**a**) Instance adding times; (**b**) Synchronous, asynchronous and history query times; (**c**) CPU usage with different consumer count; (**d**) CPU usage with different history configurations; (**e**) Memory usage in the different scenarios.

For the tests, the context manager was running on an Intel Quad-Core i5 at 1.60 GHz and with 8 GB of memory, running Windows 7 Professional Edition and JDK 1.6, both 64 bits. The service and the mobile application were connected to it through a LAN network. The CPU and memory usage statistics were collected using the JConsole [22] utility provided by the JDK. Memory statistics shown in the graphs represent the Heap Memory usage [22]. And both context adding times and query answer times presented in the graphs are measured in the clients (service and mobile application), including therefore HTTP transport delay (except for the history adding time of Figure 5(a), as this operation is performed by the context manager itself and thus is not an HTTP request). For better comprehension, statistics shown in the graphs are smoothed using linear or polynomial regression.

In Figure 5 system performance and resource demand from the defined test scenarios are presented. The first conclusion that can be drawn is that context information adding time does not noticeably depend on the number of instances already existing in the context space (see Figure 5(a)). This is true for both current context and context history. However, in the scenarios where asynchronous consumers are involved, context adding times significantly increase as a function of the already existing number of instances. This is due to the context subscription checking operation being part of the adding operation transaction. And as it can be observed, adding time also depends on the number of subscribers present on the scenario, as for every adding transaction each of the subscription queries must be checked.

In Figure 5(b) the synchronous query time for both current context and context history can be observed. Both query answering times increase as a function of the existing number of instances, tough this increase is not very pronounced. And as it would be expected, queries to the historical repository are slightly slower, as they involve accessing persistent storage. On the other hand, subscription answering delays, that is, the time elapsed since a context source provides an information item until a subscriber receives it, are also shown in this graph. It can be noticed that this delay depends both on the existing instance count and the number of subscriptions registered, as it happens for the context adding times when context subscribers are present.

Regarding processor load (see Figure 5(c)), it can be noticed that, when no consumers are present CPU demand does not depend on the instance count existing in the context space. This makes sense, as nor does context adding time depend on this fact. On the other hand, as would be expected, CPU demand increases as the number of concurrent consumers does so. And also can be observed that synchronous consumers are less CPU-demanding that asynchronous ones. This is due to each adding operation firing the subscription checking, and also because each subscription query checking involves additional tests to determine that the subscription is matched by the new added piece of information and not due to an already existing one. On to other terms, in Figure 5(d) CPU demand differences between different context history settings can be observed. As it would be expected, a context adding operation with history enabled with filtering is more demanding than invoking this method with history enabled without filtering, and the latter requires more computing power than when history is disabled. However, the differences are really subtle and it can be determined that enabling context history has not a significant impact on overall system performance.

Memory usage statistics in Figure 5(e) show a similar pattern than previous graphs; context subscribers are the most demanding entities. This way, it can be observed that the scenario where no consumers are present and the ones in which synchronous ones appear (both current context and history consumers) have very similar memory demands. This demand, even if increases together with a rise in the number of instances in the context, does it at a relatively low pace. However, when asynchronous consumers are present, memory demand is significantly higher, due to the context space snapshots kept for checking that a subscription is only fired once for the same context information item.

**Figure 6.**
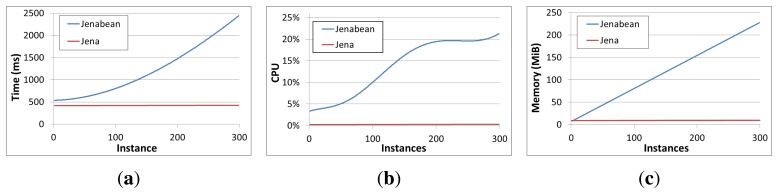
System performance in reasoning—Jena and Jenabean comparison. (**a**) Adding times; (**b**) CPU Usage; (**c**) Memory Usage.

Finally, reasoning was evaluated repeating A, B and C scenarios. In these new tests Jena OWL reasoner was used for the current context space in an on-line reasoning approach. That is, every context item added by a provider fired the reasoner. The first conclusion drawn was that as the instance number in the context space increases, system gets excessively overloaded even for scenario A, where only a context source is interacting with the context manager. After making several checks, we concluded that it was due to Jenabean library adding additional load to the reasoning process. In Figure 6 performance differences between Jenabean and Jena can be observed for the scenario A with reasoning enabled. This way, Jenabean is about five times slower in adding context information with just 300 alert instances, than Jena-only-based approach, which indeed shows almost no adding time increase in the hole test. Jenabean is also demanding almost a 25% of CPU time at the end of this test, compared to the less than 1% consumed by Jena, and requires almost ten times more memory. Therefore for completing the reasoning tests only Jena was used.

**Figure 7.**
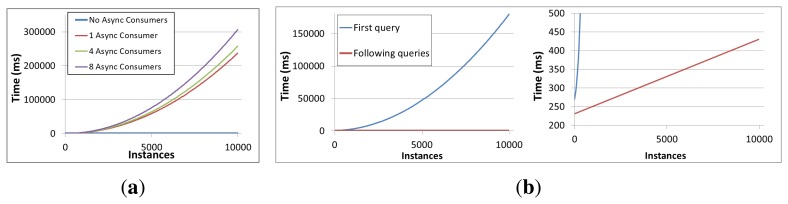
System performance in reasoning—Instance Adding and query times with Jena. (**a**) Adding times with different asynchronous consumer count; (**b**) Query times—First query after adding context and following queries.

The results of these tests can be examined in Figure 7(a). As advanced by the previous results in the Jena and Jenabean comparison, in Figure 7(a) it can be observed that as far as no context consumers are present, the adding operation is rather stable for different number of instances in the current context even when reasoning is enabled. However in scenarios where asynchronous consumers are present, adding operation time noticeably increases as a function of the existing number of instances in the system. In fact, this increase is not linear, as in the tests where reasoning is disabled, but exponential. This is due to queries being significantly slower when the reasoner is enabled as the number of instances in the context space increases. More specifically, as it can be observed in in Figure 7(b), this delay is due to the first query performed after adding context information being remarkable slower, as Jena performs reasoning work before actually answering the query.

Summing up, the tests carried out show that the system has a reasonable performance. Adding up to 60,000 triples and even having eight sources and eight synchronous consumers concurrently working against the context manager, adding operation delay keeps steady around 400 ms. Synchronous query delay also remains stable, around 200 ms for each. Context history adding time is also almost constant, being around 200 ms too. Processor and memory loads are constrained as well, with 5% CPU time and 40 MiB memory consumption in the most demanding case. However, when context subscribers are present resource demand is slightly higher. As each context addition operation causes the subscriptions to be checked, the delay of this operation depends to a greater extent on both the number of subscribers and the number of instances. Thus, adding delay increases up to 900 ms in the most demanding case (8 subscribers and 10,000 instances in the context space) and asynchronous query delay up to 600 ms. CPU and memory loads also increase as far as 11% and 375 MiB respectively.

On the other hand, the reasoning tests have shown that on-line reasoning is only feasible for a very limited number of instances in the context space, because as this figure increases, query delays increase exponentially. Therefore, reasoning should be moved away from the current context component to other entity devoted to this task.

## 5. Conclusions

In this article we have presented a context management infrastructure for mobile environments. The main goal is to enable application and services, both executed on mobile terminals or in the network, to adapt their behaviour to better meet end-user needs. For this purpose, the presented infrastructure is responsible for managing the life-cycle of the context information in the environment, assuring that it is available for these application and services to successfully carry out their tasks. Taking into account the special requirements of mobile environments, we define as key aspects of our proposal offering generic and abstract methods to work with context information, allowing future operation in different scenarios. We also rely on semantic technologies and open standards, trying to offer a solution as interoperable and extensible as possible.

On to other terms, considering that the main purpose of the infrastructure is to give service to resource limited devices like mobile phones, a context server approach is adopted. This way, the infrastructure revolves around the role of the context manager, a central context repository which relieves the more power-limited devices, such as mobile phones, from carrying out demanding tasks with context information. This central element exposes a generic interface to provide an request context information, and thus enables mobile devices to only assume this roles, and delegate to it all the demanding tasks derived from working with this kind of information. The presence of the context manager has also other advantages in terms of easing context information access for context consumers. As this central element stores all the context information provided by the sources, consumers can adopt a data-centric approach, in which they only need to worry about what information they need, not which providers supply it. And this also enables context sources to share their context information without needing to expose a querying endpoint.

To assess the validity of our proposal, we have developed a prototype of the context manager, as well as a contextual service and an end-user mobile application. With those three, we have implemented the demonstration scenario described in this paper, which shows the capabilities of the context management infrastructure. Finally, and taking this demonstrative scenario as starting point, we have carried out a series of performance tests. Both the scenario and the test suite have shown a functional system, which complies with the defined requirements and with a reasonable resource demand, being the most resource-draining cases context subscriptions and reasoning.

Therefore, our next working steps will involve improvements for these two functionalities. The current implementation of the context subscription feature implies that for every context adding transaction all the subscriptions must be checked. Similarly, reasoning is only considered in an on-line approach, in which every adding operation fires the reasoner. Thus, considering alternative policies for these features, in which they are not fired so frequently, would significantly improve system performance.

On to other terms, privacy policies have not been considered so far. In these kind of systems, where private and confidential data is flowing through different distributed entities, security measures that both prevent unauthorized access to personal data and enable legitimate access to those entities which need it to successfully carry out their tasks is essential. Consequently, next steps must take these aspects into account so as to achieve a solution that is ready for real-world deployment.

## Figures and Tables

**Listing 1.**
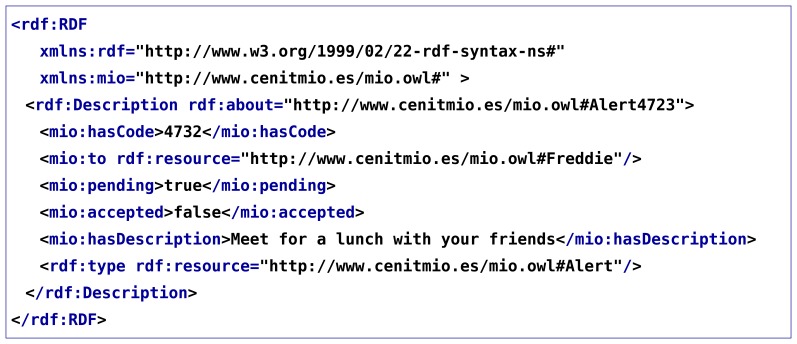
Alert instance to add to current context

**Listing 2.**
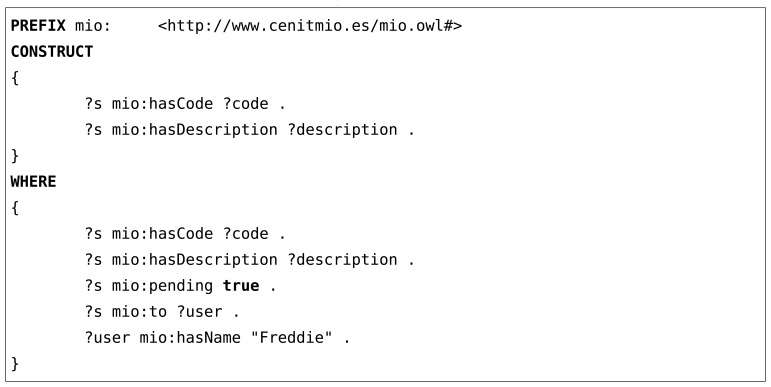
SPARQL query for retrieving alerts for a user
